# Mouse mammary tumor-like virus (MMTV) is present in human breast tissue before development of virally associated breast cancer

**DOI:** 10.1186/s13027-016-0113-6

**Published:** 2017-01-04

**Authors:** Teiko Nartey, Chiara M. Mazzanti, Stella Melana, Wendy K. Glenn, Generoso Bevilacqua, James F. Holland, Noel J. Whitaker, James S. Lawson, Beatriz G.T. Pogo

**Affiliations:** 1 Icahn School of Medicine at Mount Sinai, New York, NY USA; 2 Department of Pathology, University of Pisa, Pisa, Italy; 3 School of Biotechnology and Biomolecular Sciences, University of New South Wales, Sydney, Australia

**Keywords:** Benign breast tissues, Breast cancer, Mouse mammary tumor virus, MMTV, Mouse mammary tumor-like virus, MMTV-like, Human mammary tumour virus, HMTV, Morphology, Morphological, Histotype, Histological

## Abstract

**Background:**

There is substantial evidence that a virus homologous to mouse mammary tumor virus (MMTV) may have a role in human breast cancer. The present study indicates that those who developed breast cancer associated with an MMTV-like virus had this virus in their non-cancerous breast tissues years before the cancer developed.

**Methods:**

Polymerase chain reaction (PCR) techniques and sequencing were used to identify MMTV-like envelope gene sequences (MMTV-like *env* sequences) in Australian benign breast biopsy specimens from women who several years later developed breast cancer. Murine contamination was excluded by stringent laboratory procedures, and the absence of intracisternal A particle sequences and mitochondrial cyclooxygenase sequences.

**Results:**

MMTV-like *env* sequences (also called HMTV sequences to denote their source) were found in 9 of 25 breast cancer specimens (36%). Among 25 non-cancerous breast biopsies of these same patients taken 1 to 11 years earlier, six contained MMTV-like sequences (24%). Five of the six were among the nine virally-associated breast cancers. In two pairs of specimens, benign and malignant, *env* sequences were 97% identical.

**Conclusions:**

The identification of MMTV (MMTV-like) sequences in breast tissues prior to the development of MMTV positive breast cancer fulfills a key criterion for a possible causal role for the MMTV-like virus in human breast cancer.

## Background

Mouse mammary tumor viruses (MMTV) have a well-documented causal role in mouse mammary tumors in feral and experimental mice [1]. This role of MMTV was first observed by John Bittner in 1936 [2]. These observations led to the search for a similar virus in human breast cancer. Close morphologic and immunologic similarity were repeatedly shown. More recently, near identity of molecular structure to MMTV (90-98%) has been identified in human breast cancers [3]. The MMTV-like virus in humans is also called human mammary tumor virus (HMTV) [4].

The evidence of a role for an MMTV-like virus in human breast cancer is substantial but incomplete. An essential criterion to establish a causal role of an infectious agent in any disease, including cancer, is evidence of prior infection by the suspect agent [5]. This criterion had not been investigated with respect to MMTV-like virus. MMTV-like *env* sequences have rarely been identified in benign breast tissues [3, 6, 7], nor in the benign breast tissue of individuals with breast cancer containing MMTV-like sequences [8]. For this reason we sought to identify MMTV-like *env* sequences in benign breast biopsy tissues from women who some years later developed MMTV-like positive breast cancer.

The evidence suggestive of a role for an MMTV–like virus in human breast cancer is as follows: (i) a meta-analysis of 22 studies concluded that the identification of MMTV–like gene sequences in breast cancer tissues, was associated with a 15 fold increase in breast cancer [9], (ii) MMTV-like *env* gene sequences were identified in 38% of US human breast tumors but were extremely uncommon in healthy breast tissues [3, 6], (iii) the near complete proviral structure of MMTV-like virus that was 95-98% homologous to MMTV has been identified in human breast tumors [4, 10], (iv) MMTV viral proteins have been identified in human breast cancer [11], (v) Wnt-1 oncogene expression is significantly higher in MMTV-like positive compared to MMTV-like negative breast cancer specimens, which parallels high Wnt-1 expression in MMTV positive mouse mammary tumors [12, 13], (vi) MMTV can infect human cells and randomly integrate its genomic information [14, 15], and produce virus particles [16] (vii) there is increased prevalence of MMTV-like viral sequences in healthy breast tissues (nil), healthy tissue adjacent to breast cancer (19%), breast hyperplasia (27%), ductal carcinoma in situ (82%) [17], (viii) the age standardized rates for breast cancers in five countries of Asia are less frequent (29–43 per 100,000) than in seven countries of Europe, the Americas, and Australia (47–92) [18]. These findings correlate with different burdens of MMTV-like infection in human breast cancers, 0-20% in Asia vs. 27-60%, in the seven countries which are associated with different prevalence of MMTV in the indigenous mouse species [18, 19]. (ix) MMTV–like sequences have been identified in milk from healthy lactating women and three fold positivity in milk from women at high risk for breast cancer [20, 21], (x) MMTV-like sequences have been identified in the saliva of 27% of healthy children, 11% of healthy adults and 57% of adults with breast cancer, which is suggestive of a human to human viral transmission [22] and (xi) MMTV-like viral sequences have been identified in breast cancers which developed in a father, mother and daughter of the same family which is suggestive of an infectious condition [23]. Overall this evidence is consistent with MMTV having similar influences in both human breast cancer and mouse mammary tumors.

If an MMTV-like virus infects human breasts, MMTV-like antibodies should be present in the sera of infected individuals since mice infected with MMTV develop high titers of MMTV specific antibodies. Indeed, MMTV antibodies in human breast cancer have been described [24, 25]. Antibodies were not identified in one recent study, however [26].

## Methods

### Ethics

This project was formally considered and approved by the Human Research Ethics Committees of the several participating institutions.

Twenty five patients were identified for whom both benign breast and subsequent breast cancer specimens were available from the archives of an Australian pathology service (Douglass Hanly Moir – Pathology). All the specimens were formalin fixed and paraffin mounted. Seventeen of these sets of specimens were analysed by polymerase chain reaction (PCR) techniques at the Icahn School of Medicine at Mount Sinai (ISMMS) (New York). Eight additional specimen pairs were analysed by the same PCR techniques but with the cancer tissues microdissected from the specimens at the University of Pisa (UP) (Italy). Eight of the sets of specimens were analysed in both Centers.

### Detection of MMTV-like env sequences

The DNA extraction and detection of MMTV-like *env* sequences were performed by PCR techniques as described by Wang et al. [3]. The primer sequences used in these PCR analyses include part of the MMTV *env* gene, which differs from human endogenous retrovirus 10 (HERV-K10). The same PCR techniques were used in both the ISMMS and UP laboratories with the exception of microdissection of the tumor tissues, that were analysed in the UP laboratory by fluorescence nested PCR. Materials from all patients were not available due to the exhaustion of the blocks. The outcomes from each of the two laboratories are shown in Table 1.

**Table 1:** MMTV-like *env* gene sequences in benign breast tissues and subsequent breast cancer in the same patients

Patient	Age	Diagnosis	MMTV-like New York (ISMMS) by PCR	MMTV-like Pisa (UP) by PCR
1	36	Benign	neg	neg
	41	IDC- NST	neg	neg
2	72	Benign		neg
	75	DCIS- mucinous		neg
3	33	Benign	neg	neg
	44	IDC/DCIS- NST	neg	neg
4	45	Benign	pos	neg
	46	DCIS-cribriform	pos	neg
5	50	Benign	pos	neg
	60	ILC-pleomorphic	pos	pos
6	62	Benign	neg	
	66	DCIS- micropapillary	neg	
7	47	Benign	neg	
	56	IDC-NST	neg	
8	49	Benign		neg
	52	IDC- NST		neg
9	46	Benign	neg	
	53	IDC-NST	pos	
10	48	Hyperplasia	pos	
	52	IDC-NST	pos	
11	35	Benign		neg
	46	IDC		neg
12	44	Benign	neg	neg
	48	IDC-NST	neg	neg
13	67	Benign		neg
	75	IDC-NST		neg
14	48	Benign	neg	neg
	54	IDC-NST	neg	neg
15	42	Benign		neg
	49	IDC-NST		pos
16	42	Benign		neg
	48	IDC-NST		neg
17	39	Benign	neg	
	45	ILC	neg	
18	53	Benign	neg	
	62	DCIS	pos	
19	65	Benign		pos
	67	IDC-NST		neg
20	39	Benign	neg	
	44	DCIS-comedo	neg	
21	55	Benign		neg
	62	IDC-NST		neg
22	37	Benign	neg	neg
	39	IDC-cribriform, mucinous	neg	neg
23	39	Benign	pos	neg
	42	DCIS-comedo	pos	neg
24	48	Benign	pos	
	54	DCIS-cribriform	pos	
25	63	Benign	neg	
	67	IDC-NST	pos	

*IDC* invasive ductal carcinoma, *ILC* invasive lobular carcinoma, *DCIS* ductal carcinoma in situ, *NST* no special type, *pos* positive, *neg* negative

Contamination is a well-known problem with PCR analyses. Therefore, all reagents were shown to be free of MMTV-like sequences before use. PCR products were tested for the presence of murine mitochondrial (MoMt) and genomic DNA to exclude contamination. The methods used were as described by Deligdisch et al. [27] and outlined below:

(i) *Detection of mouse mitochondrial DNA sequences.*
  A series of PCR analyses was conducted to detect MoMt contamination by the detection of cytochrome oxidase (*cox-2*) gene as part of the MoMt in any sample DNA in which MMTV *env* sequences were detected. The following primers for *cox-2* were used: mt5982F (5-AGACGCACCTACGGTGAAGA-3) and mt16267R (5-AGAGTTTTGGTTCACGGAA CATGA-3). The product yields an amplicon of 286 base pairs. The semi-nested PCR was done using the primers mt16115F (5-TGCCAAACCCCAAAAACACT-3) and mt16267R, which results in a 153-bp amplicon. After transfer from the gel to a nylon membrane, the amplicon was detected by hybridization with a MoMt 32P-probe (5-GAACTAGAATTGATCAGGCAT-3).
(ii) *Detection of murine intracisternal A particle long terminal repeats (IAP)*
  IAPs are retrotransposon sequences present at the level of approximately 1000 copies of varying length per mouse genome. Amplification of the IAP sequences was carried out in PCR reactions using the following primers: forward primer (5-ATAATCTGCCGCATGAGCCAAGG-3) and reverse primer (5-AGGAAGAACACCACAGACCAGA-3) one cycle of 95 °C for 5 min, 35 cycles of 95 °C for 30 s, 58 °C for 30 s, 72 °C for 20 s, and one cycle of 72 °C for 7 min. If present, the products of variable size, reflecting diversity of the IAP sequences, can be visualized on a 2% ethidium bromide stained agarose gel.

## Results

The results from each of the two laboratories are shown in Table 1. Gaps in the results are due to lack of materials due to exhaustion of the blocks. The time between the benign breast biopsy specimen and subsequent breast cancer in the same patients varied from 1 to 11 years.

### Outcomes of PCR analyses

MMTV-like *env* gene sequences were identified in 6 (24%) of 25 benign breast specimens and 9 (36%) of 25 breast cancer specimens. Of the 6 MMTV-like positive benign specimens, 5 later developed (MMTV-like positive) cancer.

Eight sets of benign and later breast cancer blocks were analysed in both the ISMMS and UP laboratories. Negative outcomes of PCR analyses were the same for 5 sets of blocks. MMTV sequences were identified by both laboratories in breast cancer of patient 5. MMTV sequences were identified by the ISMMS lab only (not the UP lab) in patients 4 and 23 breast cancer specimens.

Neither MoMt nor IAP DNA sequences were identified in any of the MMTV-like *env* positive DNAs. This indicates there was no murine DNA contamination.

### Comparison of MMTV env gene sequences in benign and breast cancer specimens within the same patient

**Fig. 1:**
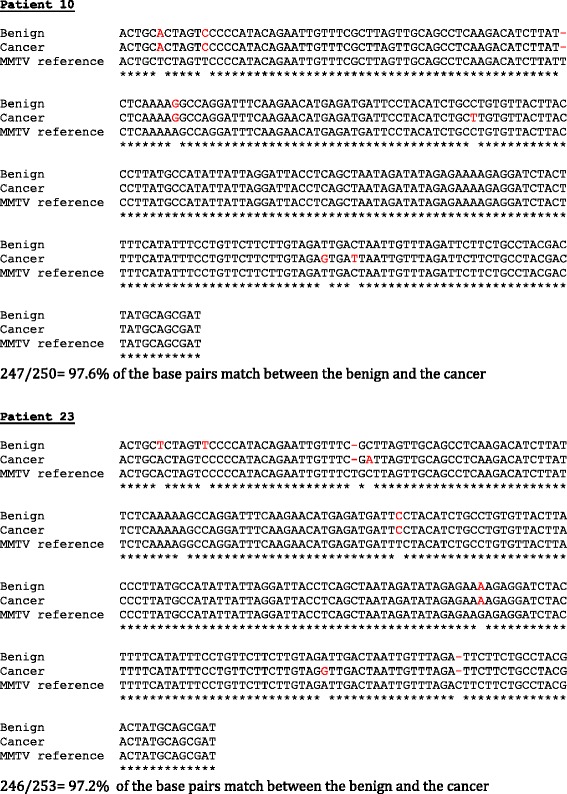
MMTV *env* gene sequences identified in benign breast and subsequent breast cancer – sequence sets of two of the same patients, compared to the MMTV (HMTV) envelope sequence DQ925473. Letters in red signify a change to the reference sequence

Over 97% of the MMTV-like *env* were identical in both the benign breast biopsy and subsequent breast cancer in 2 selected patients. These sequences are shown in Fig. 1. There are variations in approximately 3% of the sequences between the benign and subsequent breast cancers that developed several years later in the same patients. Such variations could be due to alterations in the MMTV-like genome following integration into the human genome as has been described for murine retroviruses [28] or could also occur during the PCR cycling procedures. These sequence variations indicate that contamination during PCR analyses was unlikely.

**Fig. 2:**
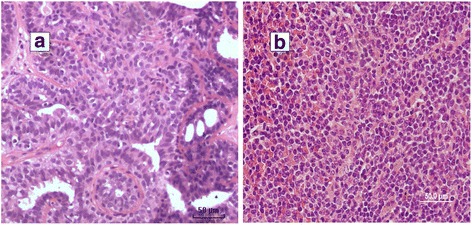
**a**. MMTV positive human breast cancer. **b**. MMTV positive mouse mammary tumour

The morphological (histotype) characteristics in 8 of the 9 MMTV-like sequence positive breast cancers were similar to MMTV positive mouse (C3H strain mice) mammary tumours. These characteristics are shown in Fig. 2.

## Discussion

In this study MMTV-like *env* gene sequences have been identified in benign breast biopsy specimens prior to the subsequent development of MMTV-like *env* positive breast cancer specimens in the same patient. This is consistent with prior infection by MMTV-like virus in breast tissues prior to the development of the same MMTV-like virus positive breast cancer some years later. This finding fulfils one key evidentiary criterion that MMTV-like virus may have a role in some human breast cancers.

## Validity of the data

We consider the data generated in this study to be valid for the following reasons: (i) The study was conducted by PCR with primers based on MMTV-like *envelope* gene sequences as described by Wang et al. [13]. These MMTV *env* gene sequences are unique to the MMTV genome and are not present in human endogenous retrovirus sequences (HERV) and which are commonly identified in studies of the human genome. (ii) Neither MoMt nor IAP DNA sequences were identified in any one of the MMTV-like *env* positive DNAs. This indicates there was no murine DNA contamination. (iii) Variations in approximately 2% of the MMTV *env* sequences of two specimen pairs were identified. This is an indication that contamination during PCR analyses was unlikely. Such variations could be due to alterations in the MMTV genome following integration into the human genome. Variations in sequences can also occur during PCR cycling procedures.

There was one case (case 19 – Table 1) where the earlier biopsy was positive for MMTV-like sequences and the ensuing breast tumor (developing after two years) was negative. In addition the results for cases 4 and 23 are not consistent between the two laboratories. There are several possible reasons: (i) MMTV sequences identified by PCR can be inconsistent and false negatives are possible; (ii) MMTV sequences may be present in some, but not all parts of the tumor; (iii) although unlikely, changes in MMTV *env* sequences may have occurred. The problem of inconsistent outcomes of PCR based analyses has been considered in detail by Vinner et al. [29]. There are particular difficulties in obtaining consistent outcomes from PCR analyses of retroviruses when present in extremely low viral concentrations. While these problems, including the exhaustion of several of the materials, do not invalidate the identification of MMTV in this current study, it would be wise to replicate this study with increased numbers of patients.

It is of interest that MMTV sequences from the long terminal repeat (LTR) section of the MMTV genome have been identified in human breast cancers using Next Generation massive parallel Sequencing (NGS) [30]. These MMTV sequences were highly homologous to the reference MMTV genome based on BLAST technology [31]. These data, based on techniques very different from PCR, confirm the identification of MMTV-like gene sequences in human breast tumors and add validity to PCR based studies that were used in this current investigation. NGS techniques are not as sensitive for the identification of retroviral nucleotide sequences as PCR [29]. This is the reason for the much more frequent identification of MMTV-like nucleotide sequences by PCR compared to NGS.

The identification of MMTV-like *env* gene sequences in 9 (36%) of 25 Australian breast cancer specimens is a similar percentage to previous investigations of Australian breast cancers [7].

It is of considerable interest that the morphology (histotype) of 8 of 9 MMTV-like positive breast cancers was similar to the morphology of MMTV positive mouse mammary tumors. This has been previously observed by Wellings [32] and Lawson et al. [33]. If this observation is confirmed this has important implications as it provides potential evidence that MMTV infections may lead to a specific morphological type of breast cancer.

## Conclusions

The aim of this project has been achieved, namely the identification of MMTV-like sequences in benign breast biopsy tissues from women who several years later developed MMTV-like positive breast cancer. The findings in this study offer an important contribution to the overall body of evidence, which links MMTV-like virus (also called HMTV) to human breast cancer and fulfills a key criterion for a possible causal role for the MMTV-like virus in human breast cancer. Although other viruses and bacteria have been found in human breast cancer, none other is known to have a similar parallel in the animal kingdom. Conclusive proof of HMTV (MMTV-like virus) as a cause of human breast cancer will open a new era for prevention and therapy [34].

## Abbreviations

BLAST technology: Basic Local Alignment Search Tool is an algorithm for comparing primary biological sequence information
*cox-2*: cytochrome oxidase gene
*env* sequences: Envelope gene sequences
HERV-K10: Human endogenous retrovirus
HMTV: Human mammary tumor virus
IAP: Intracisternal a particle long terminal repeats
ISMMS: Icahn School of Medicine at Mount Sinai (ISMMS) (New York)
LTR: Long terminal repeat section of the MMTV genome
MMTV: Mouse mammary tumor virus
MoMt: Murine mitochondrial DNA
NGS: Next Generation massive parallel Sequencing
PCR: Polymerase chain reaction
UP: University of Pisa, Italy
