# Erratum to: Mouse mammary tumor-like virus (MMTV) is present in human breast tissue before development of virally associated breast cancer

**DOI:** 10.1186/s13027-017-0126-9

**Published:** 2017-03-15

**Authors:** Teiko Nartey, Chiara M. Mazzanti, Stella Melana, Wendy K. Glenn, Generoso Bevilacqua, James F. Holland, Noel J. Whitaker, James S. Lawson, Beatriz G. T. Pogo

**Affiliations:** 1 Icahn School of Medicine at Mount Sinai, New York, NY USA; 2 Department of Pathology, University of Pisa, Pisa, Italy; 3 School of Biotechnology and Biomolecular Sciences, University of New South Wales, Sydney, Australia; 4 Fondazione Pisana per la Scienza, Pisa, Italy

## Erratum

After publication of this article [1], it was noticed the affiliation for Chiara M. Mazzanti was incorrectly included as “Department of Pathology, University of Pisa, Pisa, Italy.” The correct affiliation for Chiara M. Mazzanti is “Fondazione Pisana per la Scienza, Pisa, Italy” and has been included in this erratum in the author list and author details section.
